# Strigolactones modulate jasmonate-dependent transcriptional reprogramming during wound signalling in *Arabidopsis*

**DOI:** 10.1007/s13353-025-01005-y

**Published:** 2025-09-09

**Authors:** Marek Marzec

**Affiliations:** Faculty of Natural Sciences, Institute of Biology, Biotechnology and Environmental Protection, University of Silesia in Katowice, 40-032 Katowice, Poland

**Keywords:** *Arabidopsis thaliana*, Hormone crosstalk, Jasmonic acid, Mechanical wounding, Strigolactones, Transcriptional reprogramming

## Abstract

Mechanical wounding triggers rapid transcriptional and hormonal reprogramming in plants, primarily driven by jasmonate (JA) signalling. While the role of JA, ethylene, and salicylic acid in wound responses is well characterised, the contribution of strigolactones (SLs) remains largely unexplored. Here, for the first time, it was shown that SLs modulate wound-induced transcriptional dynamics in *Arabidopsis thaliana*. Using transcriptome profiling of wild-type (Columbia-0) and the SL biosynthesis mutant *more axillary growth3* (*max3*), a discrete cohort of genes whose wound induction is SL-dependent was identified. These genes include core JA biosynthetic genes and several JA-responsive transcriptional repressors, indicating that SLs potentiate early JA signalling. Promoter motif and protein–protein interaction analyses revealed that SLs regulate a transcriptional module composed of AP2/ERF, WRKY, and C2H2 zinc-finger factors, which integrate JA signalling, ROS homeostasis, and tissue regeneration. Notably, many of these factors are misregulated in *max3* even prior to wounding, suggesting a primed but hypo-responsive state. Presented findings suggest a model in which SLs act upstream of the JA burst, coordinating transcriptional readiness and post-injury activation. This expands the functional scope of SLs in stress response and positions them as potential modulators of hormone crosstalk during wound responses.

**Supplementary Information:**

The online version contains supplementary material available at 10.1007/s13353-025-01005-y.

## Introduction

Plants are exposed to mechanical wounding and herbivore attacks, both of which can compromise tissue integrity and trigger a defence response. Although these two stimuli share overlapping downstream responses, they differ significantly in their initial recognition mechanisms (De Bruxelles and Roberts 2001). Mechanical wounding causes physical tissue disruption, while herbivory involves both mechanical injury and the introduction of herbivore-associated molecular patterns (HAMPs) from insect oral secretions or oviposition fluids (Gandhi et al. 2020; Reymond 2021).

Perception of wounding starts at the cell surface via pattern recognition receptors (PRRs), which detect damage-associated molecular patterns (DAMPs), microbe-associated molecular patterns (MAMPs), or HAMPs, leading to the activation of pattern-triggered immunity (PTI) or effector-triggered immunity (ETI) (Sun et al. 2018; Sun and Zhang 2021). These signalling events trigger rapid depolarisation of plasma membrane potential, followed by calcium ions (Ca^2+^) influx, which activates a wide array of downstream responses (Camoni et al. 2018; Gandhi et al. 2020). The elevation of cytosolic Ca^2+^ is decoded by Ca sensors such as calmodulins (CaMs), which then engage transcriptional regulators and kinase cascades (Aldon et al. 2018; Kudla et al. 2018).

A hallmark of the wounding response is the activation of jasmonic acid (JA) biosynthesis. This is triggered by Ca^2^⁺-dependent kinases that phosphorylate JASMONATE-ASSOCIATED VQ-MOTIF GENE 1 (JAV1), disrupting the JAV1–JASMONATE ZIM-DOMAIN PROTEIN 8) JAZ8–WRKY51(JJW) complex and promoting JA accumulation (Yan et al. 2018). JA and its bioactive conjugate JA-Ile bind to the (CORONATINE-INSENSITIVE PROTEIN 1) COI1 receptor, initiating degradation of JAZ repressors and allowing activation of defence-related transcription factors (TFs) (Paschold et al. 2008; Wasternack and Hause 2013). Reactive oxygen species (ROS), are also rapidly produced and serve as local signals and systemic cues(Savatin et al. 2014; Prasad et al. 2020). Furthermore, nitric oxide (NO), a reactive nitrogen species, contributes to wound signalling and can modulate both JA biosynthesis and ROS production (Delledonne et al. 1998; Leitner et al. 2009). Systemic signalling enables communication between the damaged site and distal tissues. Ca^2^⁺ waves, JA, and its precursors such as 12-oxo-phytodienoic acid (OPDA) are transported through the phloem or synthesised de novo in distal tissues (Taki et al. 2005; Koo et al. 2009). Salicylic acid (SA) often antagonises JA, particularly in pathogen responses, but recent studies suggest it also fine-tunes wound-induced defences. For example, *Arabidopsis* mutants with disrupted SA signalling exhibit altered ROS dynamics and compromised wound responses (Ogawa et al. 2010). Ethylene, another key hormone, synergizes with JA to amplify defence gene expression, as seen in tomato (*Solanum lycopersicum*) plants where ethylene-insensitive mutants show reduced protease inhibitor production (Díaz et al. 2002).

At the transcriptional level, mechanical wounding induces massive reprogramming. Microarray analyses in Arabidopsis revealed that ~ 8% of the transcriptome changes within 30 min after wounding, including strong activation of transcription factors (e.g., WRKY, MYB, AP2/ERF), calcium-related signalling proteins, and receptor-like kinases (Cheong et al. 2002). Several of these genes are also involved in pathogen responses, suggesting substantial overlap between wound and pathogen defence signalling (Maleck and Dietrich 1999; Durrant et al. 2000). Interestingly, many wound-responsive genes are regulated in a JA-dependent manner, yet a significant number remain inducible in JA-insensitive mutants, indicating the existence of parallel JA-independent pathways (Reymond et al. 2000). Moreover, feeding by *Pieris rapae* larvae triggers transcriptional profiles distinct from those induced by mechanical wounding alone. Also, some water-stress-related genes activated by wounding are suppressed during herbivory, suggesting that insects may suppress or manipulate plant defence (Reymond et al. 2000).

Despite extensive research into the roles of JA, SA, and ethylene in wound signalling, little is known about the contribution of strigolactones (SLs), a class of carotenoid-derived phytohormones known for regulating shoot architecture, root development, and stress adaptation. Given their regulatory roles and potential to interact with other hormonal pathways (Korek et al. 2025), as well as their role in ROS homeostasis (Daszkowska-Golec et al. 2023), SLs may influence the transcriptional and physiological responses triggered by wounding. In this study, the potential role of SLs in the regulation of wound-induced signalling is examined. Using SL-biosynthesis mutant *more axillary growth3* (*max3*) mutant (Booker et al. 2004) and wild-type (WT) plants, the SL-dependent transcriptional response to mechanical wounding was analysed.

## Results

### Wound-induced transcriptome response in WT and *max3*

**Fig. 1:**
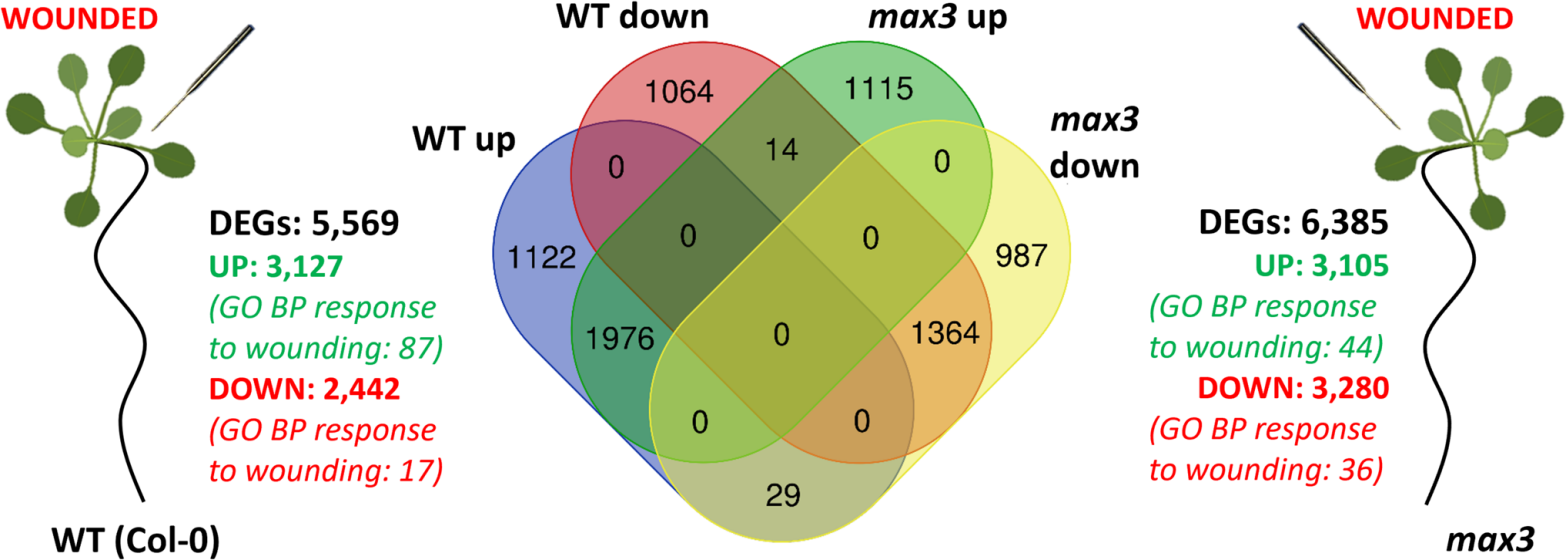
Transcriptome response to mechanical wounding in *Arabidopsis thaliana* WT (Col-0) and SL-deficient *max3* mutant. Differentially expressed genes (DEGs; log₂FC ≥ 1 or ≤  − 1; adjusted *P* ≤ 0.01) were identified 3 h after wounding. A Venn diagram illustrates the overlap between DEGs up- or downregulated in each genotype. DEGs were identified by comparing wounded and control plants within each genotype separately

In order to determine whether SLs influence the wound response in *Arabidopsis thaliana*, transcriptome profiling was performed on wild-type (Columbia-0) and the SL biosynthesis mutant *max3* (Booker et al. 2004). Plants were wounded, and gene expression was analysed three hours post-treatment. In the wild type, 5,569 genes showed differential expression (log₂FC ≥ 1 or log₂FC ≤  − 1; adjusted *P* ≤ 0.01), including 3,127 up-regulated and 2,442 down-regulated genes (Fig. 1; Table S1). In *max3*, 6,385 genes were differentially expressed, with 3,105 up-regulated and 3,280 down-regulated. Gene Ontology (GO) analysis indicated that 87 up- and 17 down-regulated genes in the wild type were annotated with the term “response to wounding.” In *max3*, 44 up- and 36 down-regulated genes were classified within this category (Fig. 1; Table S1). While a large number of DEGs were shared between genotypes, the magnitude of induction for some genes differed considerably, particularly for those related to JA biosynthesis and signalling.

### SL-dependent transcriptome response to wounding

To select wound-induced genes regulated by SLs, DEGs unique to WT were identified (Fig. 1, Table S2). This subset contained 2,186 genes (1,122 up- and 1,064 down-regulated). A comparable number was exclusive to *max3* (2,102; 1,115 up- and 987 down-regulated). The largest group, 3,340 DEGs (1,976 up- and 1,364 down-regulated), was shared by both genotypes, indicating that only part of the wound response relies on SLs, with substantial SL-independent pathways acting in parallel. Opposing regulation was rare: 29 genes were up- in WT and down-regulated in *max3*, while 14 showed the inverse pattern (Fig. 1, Table S2).

Among genes up-regulated exclusively in WT following mechanical wounding, several were previously associated with JA signalling and wound-induced responses. This group includes *AOC2* (AT3G25770, log₂FC = 2.45), *OPR3* (AT2G06050, 1.15), *DGL* (AT1G05800, 5.55), and *JAZ1* (AT1G19180, 2.60) (Table S2b). These genes encode enzymes or regulators directly involved in JA biosynthesis and signalling, or transcriptional modulators of wound-related pathways. In total, 35 genes related to JA biosynthesis or signalling, and 42 genes related to wounding response, were up-regulated exclusively in WT (Table S2b). However, transcript levels do not necessarily reflect hormone abundance, and no direct measurements of JA or JA-Ile were performed in this study. Future work involving targeted quantification of JA will be necessary to validate these inferences. In contrast, the list of genes down-regulated exclusively in WT included key photosynthesis-related genes such as *LHCB3* (AT1G29910, log₂FC = –1.42), *PSAK* (AT1G30380, –1.29), *CP29.1* (AT5G01530, –1.23), and *RPE* (AT3G01850, –1.16), encoding components of the photosystem I/II antenna and Calvin cycle (Table S2e).

However, among the 1,976 DEGs identified in WT and *max3* after wounding (Table S2a), several encode key components of the canonical JA pathway. These include the lipoxygenases LOX3 (AT1G17420; log₂FC = 4.10 in WT, 1.84 in *max3*) and LOX4 (AT1G72520; 2.92, 1.79), the chloroplast enzymes AOC3 (AT3G25780; 4.21, 3.08) and AOS (AT5G42650; 2.28, 1.64), and the JA-responsive repressor JAZ10 (AT5G13220; 3.50, 2.41). In total, 52 genes annotated with “JA biosynthetic process” or “JA signalling” and 67 genes annotated with “response to wounding” were co-induced in both genotypes. The vast majority of these genes showed up-regulation in both genotypes following wounding (Table S2a and S2e).

### Enrichment analysis of SL-dependent wound-responsive genes

GO enrichment highlighted a strong stress-related signature (Fig. S1). “Response to wounding” showed the highest fold enrichment (3.7; FDR = 2.4 × 10⁻⁸), followed by processes linked to jasmonate signalling (“response to fatty acid”, “response to jasmonic acid”) and detoxification/hypoxia. Secondary-metabolism terms, including sulphur-compound and organic-acid metabolism, were likewise over-represented, consistent with activation of specialised defence pathways. In contrast, GO terms over-represented among repressed genes were almost exclusively photosynthetic (Fig. S1). “Photosynthesis light reaction” (fold = 6.8; FDR = 1.1 × 10⁻^1^⁶), “photosystem II assembly” (9.6), and “chloroplast organisation” (4.0) dominated the list, indicating a coordinated shutdown of light harvesting, electron transport and chlorophyll metabolism in WT that is absent in *max3*. Genes associated with the enriched GO categories are listed in Supplementary Table S2, which includes GO annotations for biological processes for all DEGs.

### SL-dependent TFs involved in the response to wounding

To uncover the regulation mechanisms responsible for transcriptome response to wounding, which may be SL-dependent, the transcription factors (TFs) active only in WT were identified. First, the set of DEGs specific for WT was screened to select potential TFs. Among the 123 TFs induced exclusively in WT after mechanical wounding (Table S3), GO enrichment analysis revealed overrepresentation of the biological process term “response to wounding”. In addition, two TFs, *ERF13* (AT2G44840, log₂FC = 2.11) and *WRKY28* (AT4G18170, log₂FC = 1.87), were also identified as wound-responsive in an independent transcriptome dataset profiling Arabidopsis roots after mechanical injury (ArrayExpress E-MTAB-7609). *ERF13* expression increased at 1 h post-wounding, while *WRKY28* was consistently up-regulated at 1, 3, and 6 h, in this experiment. Several other TFs from this group have also been functionally implicated in jasmonate-dependent wound signalling. This includes *ZAT12* (*AT5G59820*, log₂FC = 2.49), *ERF5* (*AT5G47230*, 2.20), *ZAT10* (*AT1G27730*, 2.94), *MYB4* (*AT4G38620*, 1.45), *WRKY53* (*AT4G23810*, 1.81), and an uncharacterised C2H2-type zinc finger gene (*AT3G53600*, 5.18) (Table 1). *ZAT12* has been shown to undergo rapid and systemic induction following wounding, based on promoter::LUC activity in leaves (Davletova et al. 2005). *ZAT10, ERF5, WRKY53*, and *MYB4* are all JA-, MeJA-, and OPDA-inducible, and were part of the early transcriptional wave triggered by mechanical damage (Taki et al. 2005). *ERF5* contributes to JA-mediated defence against *Botrytis cinerea*, acting redundantly with *ERF6* (Moffat et al. 2012). Finally, *AT3G53600* was identified as a positive regulator of JA-mediated wound signalling in a COI1-dependent screen; its overexpression enhances JA-responsive gene expression (Wang et al. 2007).

**Table 1:** SL-dependent wound-responsive transcription factors in *Arabidopsis*

TFs identified among wound-induced DEGs exclusively in WT				
Gene ID	Gene name	TF family	log₂FC	Evidence for wound response
AT5G59820	ZAT12	C2H2	2.49	Induced by wounding, JA, MeJA, OPDA (Davletova et al. 2005; Taki et al. 2005)
AT5G47230	ERF5	ERF	2.2	Induced by wounding, JA, MeJA, OPDA (Taki et al. 2005)
AT1G27730	ZAT10	C2H2	2.94	Induced by wounding, JA, MeJA, OPDA (Taki et al. 2005)
AT4G38620	MYB4	MYB	1.45	Induced by wounding, JA, MeJA, OPDA (Taki et al. 2005)
AT4G23810	WRKY53	WRKY	1.81	Induced by wounding, JA, MeJA, OPDA (Taki et al. 2005)
AT3G53600	-	C2H2	5.18	Acts downstream of JA; over-expression enhances JA-mediated wound signalling; positive regulator in a COI1-dependent screen (Wang et al. 2007)
AT2G44840	ERF13	ERF	2.11	wounding’ at ‘1 h’ vs ‘none’ at ‘0 h’ (E-MTAB-7609)
AT4G18170	WRKY28	WRKY	1.87	wounding’ at ‘1 h’ and ‘3 h’ and ‘6 h’ vs ‘none’ at ‘0 h’ (E-MTAB-7609)
Overrepresented (OR) TFs control the expression of DEGs wound-induced exclusively in WT				
Gene ID	Gene name	TF family	Evidence for wound response	
AT1G78080	WIND1	AP2/ERF	Master regulator of wound-induced cellular re-programming; promotes callus formation and shoot regeneration after injury (Iwase et al. 2017)	
AT2G22300	CAMTA3	CaM	Mediates Ca^2^⁺/calmodulin-dependent signalling; required for herbivore- and wound-induced jasmonate accumulation and defence activation (Qiu et al. 2012)	
AT4G16150	CAMTA5	CaM	Binds the CCGCGT motif; regulates expression of ethylene-responsive genes via a promoter element characterised as wound-responsive. (Moore et al. 2022)	
AT1G74930	ORA47	AP2/ERF	positive regulator in a COI1-dependent screen (Wang et al. 2007); “leaf 8 wounded” vs “unwounded” (E-GEOD-41779)	
AT1G12610	DDF1	AP2/ERF	Rapidly induced by mechanical wounding (Morker and Roberts 2011); wounding’ at ‘1 h’ vs ‘none’ at ‘0 h’ (E-MTAB-7609); “leaf 8 wounded” vs “unwounded” (E-GEOD-41779)	
AT3G22830	-	HSF	wounding’ at ‘1 h’ vs ‘none’ at ‘0 h’ (E-MTAB-7609)	
AT5G59780	HSFB3	HSF	“leaf 8 wounded” vs “unwounded” (E-GEOD-41779)	
Common DEG and OR TFs				
AT2G41690	HSFA6B	HSF	wounding’ at ‘3 h’ vs ‘none’ at ‘0 h’ (E-MTAB-7609)	

Genes underlined were also identified under control conditions between *max3* and WT, indicating potential constitutive misregulation in the mutant

**Fig. 2:**
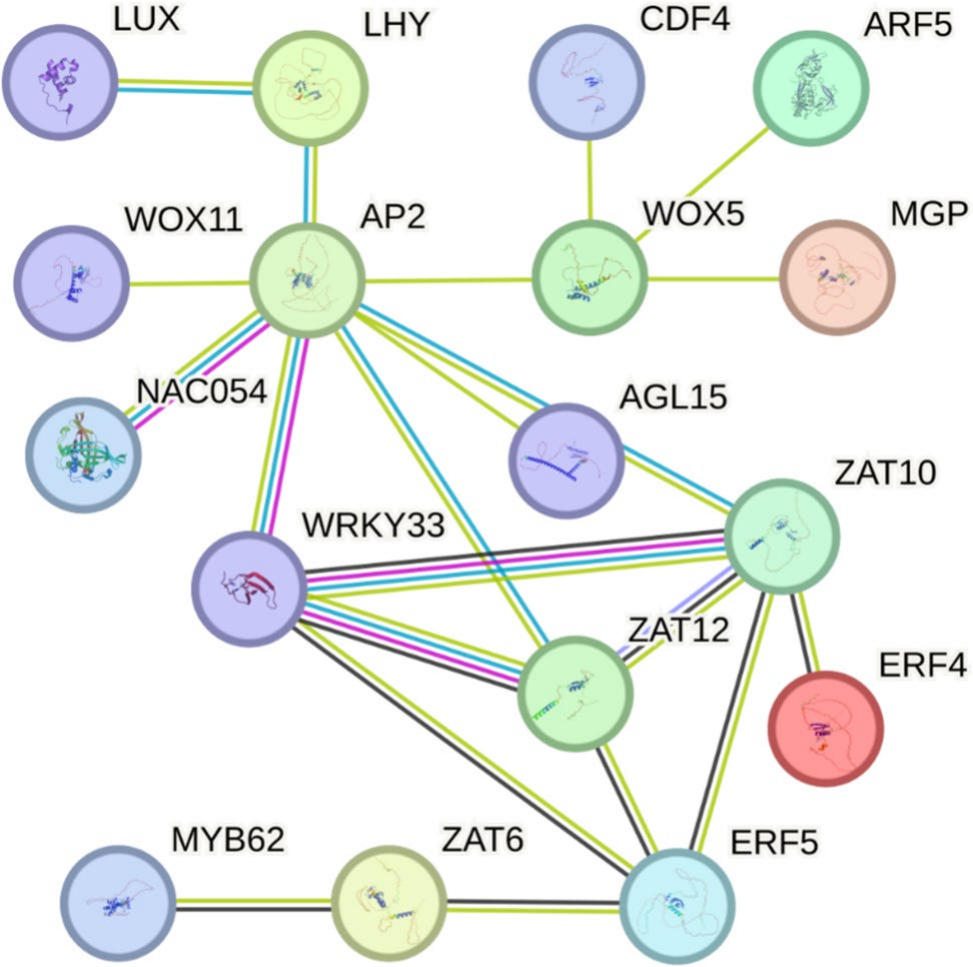
Predicted protein–protein interaction network (STRING v11.5) of TFs induced exclusively in WT plants in response to wounding. The network was generated using high-confidence interaction scores (≥ 0.7) and includes known and predicted associations based on co-expression, experimental data, and database annotations. Coloured edges represent different types of evidence

Protein–protein interaction prediction using the STRING database revealed a highly interconnected regulatory module for the identified TFs (Fig. 2), based on integrated evidence including physical binding, co-expression, and functional associations. The resulting network showed several TFs (e.g. ZAT12, ZAT10, WRKY33, ERF5, AP2) with multiple predicted connections to other regulators implicated in stress and developmental processes. Notably, ZAT10, ZAT12, and ERF5 appeared within the same cluster and showed predicted co-expression and functional linkage. The presence of *WRKY33*, a known regulator of pathogen-induced transcriptional cascades and jasmonate signalling (Zhou et al. 2015), supports the hypothesis that SLs modulate stress-responsive gene networks following wounding. Notably, *WRKY33* interacts with key JA-regulated promoters and contributes to resistance against necrotrophic pathogens, indicating a possible convergence point for SL-JA crosstalk (Zhou et al. 2015). The inclusion of developmental regulators such as *WOX5*, *LHY*, and *ARF5* (Costanzo et al. 2014; Park et al. 2016; Liu et al. 2018) suggests that wound-induced reprogramming may involve TFs that also function in stem cell maintenance (*WOX5*), circadian regulation (*LHY*), and auxin-mediated development (*ARF5*). Their predicted co-regulation with stress-responsive TFs implies that SLs might help coordinate the balance between regeneration and defence in wounded tissues.

Next, the promoter sequences (1500 bp) of each WT-specific wound-induced DEG were screened to identify the TF binding sequences. Those analyses allowed for the identification of the 19 overrepresented TFs that regulate the transcription of WT DEGs (Table S4). Among them, several have been previously characterised as wound-induced and functionally relevant to tissue damage responses (Table 1). The AP2/ERF-domain TF *WIND1* (*AT1G78080*) was known to act as a master regulator of wound-induced cellular reprogramming. WIND1 promotes callus formation and de novo shoot regeneration at wound sites, and its transcription is rapidly induced following injury (Iwase et al. 2017). Two members of the calmodulin-binding transcription activator family, *CAMTA3* (*AT2G22300*) and *CAMTA5* (*AT4G16150*), were also identified. CAMTA3 is required for wound- and herbivore-induced JA biosynthesis and defence activation (Qiu et al. 2012) (Qiu et al. 2012), while CAMTA5 binds the CCGCGT element in the promoters of ethylene-responsive genes and contributes to transcriptional activation under mechanical stress (Moore et al. 2022). Another AP2/ERF-domain TF, *ORA47* (*AT1G74930*), previously shown to function as a positive regulator of the JA pathway in a COI1-dependent manner, was also predicted as a key regulator. Its expression is induced in the wound-related transcriptome dataset (ArrayExpress E-GEOD-41779). *DDF1* (*AT1G12610*), another AP2-like factor, was up-regulated in light-dependent wound-response assays (Morker and Roberts 2011) and showed consistent induction in both root and leaf wounding datasets (ArrayExpress E-GEOD-41779, E-MTAB-7609). Additional TFs with transcriptomic evidence of wound-responsiveness include *HSFB3* (*AT2G41690*), *HSFA6B* (*AT3G22830*), and *AT5G59780*. All were identified as up-regulated in independent microarray or RNA-seq datasets profiling Arabidopsis tissues at early timepoints post-wounding (ArrayExpress E-GEOD-41779, E-MTAB-7609). Importantly, since these TFs were wound-induced exclusively in WT but not in max3, this indicates that their activation depends on a functional strigolactone pathway.

### SL-related transcriptome differences under control conditions

**Fig. 3:**
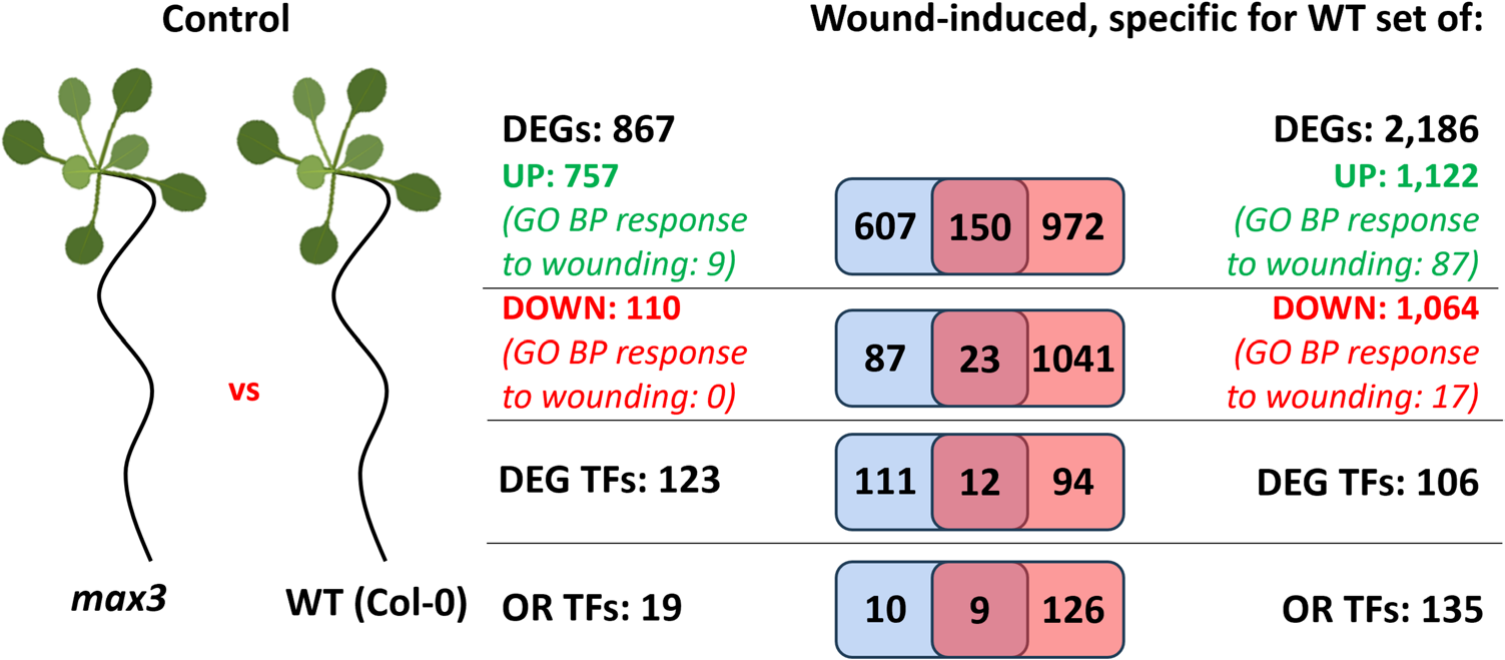
Comparative analysis of transcriptomic responses in WT and *max3* under control and wound-induced conditions. Left panel: DEGs and TFs identified in comparison of *max3* and WT under control conditions. Right panel: Wound-induced DEGs specific to WT and TFs that may control the expression of those DEGs

To determine whether the altered wound response observed in *max3* reflects pre-existing transcriptional differences rather than a failure to activate specific genes upon injury, the gene expression profiles between *max3* and WT under control conditions were compared. This analysis identified 867 differentially expressed genes (DEGs), including 757 up-regulated and 110 down-regulated in *max3* relative to WT (Fig. 3) (Table S5). Notably, 173 of these genes (19.9%) overlapped with the set of wound-induced DEGs specific to WT, indicating that a subset of SL-dependent wound-responsive genes is already misregulated in *max3* prior to wounding (Fig. 3) (Table S5). The presence of wound-inducible genes among those up-regulated in *max3* under control conditions suggests that SLs may contribute to their repression at rest, preventing premature activation in the absence of stress.

Among 867 DEGs under control conditions, 12 encoded transcription factors (Table S5e). Three of them, *AT3G53600*, *ZAT10*, and *ZAT12*, have previously been implicated in the plant wound response, supporting the hypothesis that some key regulators of the SL-dependent transcriptional programme are constitutively dysregulated in the mutant. Promoter sequence analysis of the 867 misregulated genes revealed enrichment of binding motifs for 135 transcription factors (Table S5g). Among these, 9 TFs were previously predicted to regulate wound-induced genes specific to WT (Fig. 3) (Table S5h). Notably, five of these nine TFs have documented roles in the wound response (Table 1), further supporting their potential contribution to the impaired transcriptional activation observed in *max3*.

Together, these basal and interaction analyses show that (i) *max3* seedlings are transcriptionally reprogrammed even before wounding, and (ii) a discrete cohort of genes requires SL signalling for full induction after injury, beyond constitutive differences between the genotypes.

## Discussion

### Strigolactones influence multiple levels of jasmonate biosynthesis and signalling

Previous studies indicate that SLs modulate JA signalling at both transcriptional and metabolic levels (Nagata et al. 2015; Song et al. 2020; Lahari et al. 2024; Han et al. 2025). In this study, the SL biosynthesis mutant *max3* exhibited impaired induction of canonical JA biosynthetic genes (*DGL*, *AOC2*, *OPR3*) (Hyun et al. 2008; Chini et al. 2018; Yang et al. 2023) and JA-responsive repressors (*JAZ1*) (Thines et al. 2007) following wounding, suggesting that SLs are required for the full activation of the early JA-dependent response to injury. It is important to note that these conclusions are based solely on transcriptomic data alone; no direct quantification of JA, JA-Ile, or related metabolites was conducted. Thus, the presented model should be viewed as a transcriptional hypothesis pending metabolic validation. Previous reports show that SL mutants display attenuated JA responses under stress conditions, including drought and pathogen infection (Piisilä et al. 2015; Lahari et al. 2024). In rice, SLs were shown to act as negative regulators of pathogen defence in rice by antagonising jasmonate (JA) signalling. SL mutants (biosynthesis—*d10* and signalling—*d14*) and SL-depleted plants (treated with SL biosynthetic inhibitor TIS108) showed enhanced resistance to *Pyricularia oryzae*, accompanied by elevated levels of JA, sugars, and flavonoid phytoalexins. This enhanced defence required an intact JA pathway, as JA-insensitive mutants (*jar1*, *coi1-18*) did not benefit from SL depletion (Lahari et al. 2024). Recent multi-omics studies in barley support and extend these findings. In the SL-insensitive mutant *hvd14*, 2-week-old seedlings accumulate ~ 2.6-fold more JA than WT, whereas 4-week-old mutants show a ~ 40% reduction in JA content (Korek et al. 2025). This developmental inversion of JA levels is paralleled by transcriptional upregulation of *lipoxygenase (LOX)* genes, key enzymes in the JA biosynthetic pathway. Suggesting that SL signalling affects not only gene expression but also downstream metabolic flux or stability of JA. In line with this, proteomic data from *hvd14* mutants revealed elevated levels of enzymes involved in early JA biosynthesis but no corresponding increase in late-pathway components or JA conjugates (Korek et al. 2025). These findings suggest that SL signalling influences JA-related processes at multiple regulatory levels, and that the effects may vary depending on tissue, developmental stage, or type of stress encountered. While SL mutants show impaired wound-induced JA responses in Arabidopsis, they may activate alternative defence mechanisms or accumulate JA differently under pathogen attack, as observed in rice and barley.

**Fig. 4:**
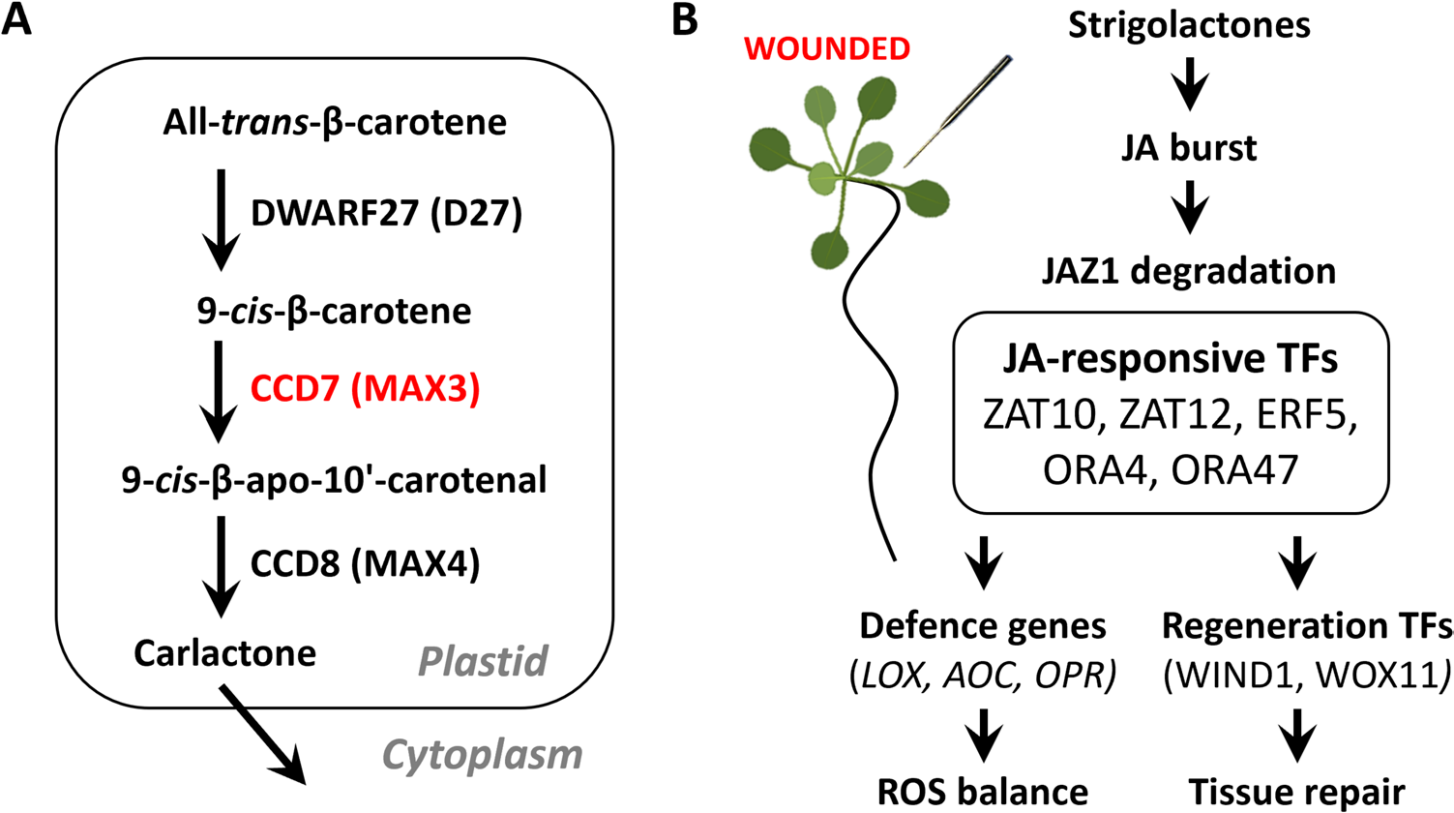
Strigolactone (SL) biosynthesis and proposed model of SL–JA crosstalk during the wound response in *Arabidopsis thaliana*. **A** Simplified scheme of the SL biosynthetic pathway. MAX3 (CCD7) is a carotenoid cleavage dioxygenase that converts 9-cis-β-carotene into 9-cis-β-apo-10′-carotenal, a key precursor of carlactone. The *max3-9* mutant used in this study lacks functional CCD7 and is therefore deficient in SL production. **B** Proposed model of SL-JA crosstalk during the wound response. Mechanical injury triggers SL-dependent activation of a jasmonic acid (JA) burst, which promotes degradation of JAZ1 (JASMONATE ZIM-DOMAIN PROTEIN 1), a key repressor of JA signalling. This releases a set of JA-responsive TFs, including ZAT10 and ZAT12 (ZINC FINGER OF ARABIDOPSIS THALIANA 10/12), ERF5 (ETHYLENE RESPONSE FACTOR 5), and ORA47 (OCTADECANOID-RESPONSIVE ARABIDOPSIS AP2/ERF 47). These TFs activate expression of genes associated with defence, including *LOX* (*LIPOXYGENASE*), *AOC* (*ALLENE OXIDE CYCLASE*), and *OPR* (*12-OXOPHYTODIENOATE REDUCTASE*), as well as genes involved in regeneration and ROS balance, such as *WIND1* (*WOUND-INDUCED DEDIFFERENTIATION 1*) and *WOX11* (*WUSCHEL-RELATED HOMEOBOX 11*). Together, these pathways promote effective tissue repair and the re-establishment of redox homeostasis following mechanical damage

Importantly, comparative transcriptome profiling under non-wounded conditions revealed that the JA network in *max3* is already perturbed before any stress is applied: 867 genes are differentially expressed relative to WT, and nearly 20% (173 genes) belong to the cohort that in WT responds specifically to wounding (Fig. 3; Table S5). Among them are core JA biosynthetic enzymes (e.g. LOX, AOC, OPR isoforms), canonical repressors (JAZ1), and key wound-responsive transcription factors (ZAT10, ZAT12, AT3G53600). Promoter motif enrichment further points to nine transcription factors with documented roles in wound signalling whose targets are mis-regulated in the mutant. Altogether, these data support a model in which SLs enhance wound-induced JA signalling by promoting biosynthetic gene induction, maintaining hormone accumulation, and possibly relieving repression by negative regulators. Our observation strengthens the hypothesis that SLs act as upstream modulators of the JA burst. For clarity, a simplified scheme of the SL biosynthetic pathway is presented in Fig. 4A, with MAX3/CCD7 highlighted.

### SLs coordinate transcriptional modules linking JA signalling, ROS homeostasis, and regenerative pathways

Promoter enrichment and protein–protein interaction analyses identified a transcriptional module regulated by SLs and centred around C2H2-type zinc-finger proteins (ZAT10, ZAT12), AP2/ERF-domain transcription factors (ERF5, ORA47, WIND1), and WRKY proteins. Several of these transcription factors are known integrators of jasmonate (JA) and reactive oxygen species (ROS) signalling (Davletova et al. 2005; Moffat et al. 2012). This supports a model in which SLs modulate early wound responses by enhancing the activity of transcriptional regulators that coordinate JA production and signalling (Fig. 4B).

SLs also appear to play an important role in maintaining ROS homeostasis under stress conditions. In barley, the SL-insensitive mutant *hvd14* exhibits increased susceptibility to drought or heavy metal stress, as reflected by severe chlorophyll loss, reduced PSII efficiency, accumulation of anthocyanins, and elevated hydrogen peroxide levels, compared to WT plants (Daszkowska-Golec et al. 2023). Application of synthetic SL analogues (GR24) or the upregulation of antioxidant enzymes (e.g., SOD, CAT) ameliorates these effects in various species (Qiu et al. 2021; Wani et al. 2023; Shah et al. 2023). Given the well-established role of ROS as upstream signals in JA biosynthesis and wound responses, impaired ROS detoxification in SL-deficient genotypes may contribute to the reduced JA accumulation observed following wounding.

In addition to defence-related signalling, several TFs in the SL-dependent module are involved in post-injury tissue regeneration. Notably, WIND1 (Iwase et al. 2015, 2017) and WOX11 (Wan et al. 2023), transcriptional regulators required for callus formation and vascular reconnection, are induced specifically in WT plants and are predicted targets of JA/ROS-associated TFs (Iwase et al. 2017). These findings indicate that SLs contribute to reprogramming processes necessary for tissue repair and developmental re-patterning following mechanical damage.

Collectively, these results indicate that SLs regulate a transcriptional programme linking hormone signalling, oxidative stress responses, and regenerative processes. This programme is activated both prior to and in response to injury, and its disruption in SL-deficient plants compromises the efficiency of JA-mediated signalling, ROS homeostasis, and tissue regeneration.

### A model for SL–JA crosstalk in wound signalling

The data support a model in which SLs act upstream of a transcriptional hub that integrates JA biosynthesis, reactive-oxygen signalling and tissue repair. In WT plants, wounding rapidly induces a set of C2H2 zinc-finger and AP2/ERF transcription factors, together with regulators of cellular reprogramming (Table 1). Promoter enrichment analysis suggests that these TFs may regulate a substantial portion of the SL-dependent wound transcriptome, including genes encoding core JA biosynthetic enzymes. Thus, it is proposed that SL signalling maintains these TFs at low basal levels while preserving their inducibility. Upon injury, SL perception relieves repression of the hub, allowing a rapid JA burst, ROS detoxification and activation of regeneration pathways.

In the *max3* mutant, this regulation is uncoupled. Nearly one-fifth of the genes mis-expressed under control conditions belong to the WT wound-responsive set, indicating partial pre-activation of the hub. However, the same TFs and biosynthetic genes fail to reach WT expression after wounding, leading to insufficient JA accumulation and an attenuated defence switch. Constitutive up-regulation of negative regulators and impaired ROS homeostasis likely feed back to restrict further induction. Thus, SL deficiency creates a “primed-but-hyposensitive” state in which defence costs are incurred without full protective benefit. This working model may explain how SLs calibrate the balance between preparedness and responsiveness in the JA pathway and clarifies why SL-deficient mutants are handicapped in acute wound signalling despite exhibiting basal stress signatures.

### Limitations and future directions

This study presents a preliminary, transcriptome-based analysis of wound-induced responses in a single SL-deficient mutant (*max3-9*) at one timepoint (3 h post-wounding). As such, the conclusions drawn here are restricted to transcriptional dynamics and cannot directly infer changes at the metabolic, hormonal, or physiological level. In particular, no quantification of endogenous SL or JA levels was performed, and functional validation via phenotypic assays (e.g. pathogen resistance) was not included. These omissions reflect the exploratory nature of the study as well as current technical constraints. Future work should include time-resolved transcriptomic and hormonal profiling, targeted JA and SL quantification, and the use of additional SL mutants to validate the generality of the observed transcriptional patterns. Combining transcriptomic data with phenotypic or biochemical outputs would also allow for the identification of functionally relevant SL-JA interactions during tissue regeneration or defence. Despite its limitations, the dataset generated here provides a useful starting point and defines a testable framework for SL-mediated modulation of wound signalling.

## Materials and methods

### Plant material, growth conditions, and stress application

The WT Arabidopsis thaliana plants used in this study belonged to the Columbia-0 (Col-0) ecotype. The SL-depleted mutant *max3-9*, lacking *CCD7* (*Carotenoid Cleavage Dioxygenase 7*) function (Booker et al. 2004), was obtained from Prof. Christine Beveridge. This line was generated in the Col-0 background. WT and mutant seeds were propagated simultaneously and grown under identical conditions prior to the experiment. Arabidopsis seeds were sterilised by immersion in 70% ethanol containing 0.5% Tween 20 for 10 min, followed by three washes in absolute ethanol and subsequent air drying. Seeds were then sown on half-strength Murashige and Skoog (1/2 MS) medium (Murashige and Skoog 1962) and grown under controlled environmental conditions (22 °C, 16 h light/8 h dark photoperiod, 200 µmol m^−2^ s^−1^ light intensity). The wounding assay was performed on 4-week-old seedlings. Each Petri dish contained a single set of plants, either wounded or unwounded, representing a specific genotype. Mechanical wounding was carried out using a 0.5 mm diameter laboratory needle, puncturing each leaf at four distinct sites. Control plates were briefly opened (2 min) to match the exposure time during wounding. All samples were subsequently returned to the growth chamber for a 3-h recovery period.

### Tissue collection and RNA isolation

Plant material (shoots) was harvested three hours after the onset of stress treatment and immediately snap-frozen in liquid nitrogen. For each biological replicate, tissue samples from four individual plants were pooled. A total of four biological replicates per genotype were prepared, each originating from a separate plate. Frozen samples were ground in liquid nitrogen using a mortar and pestle. Total RNA was extracted using the mirVana™ miRNA Isolation Kit (ThermoFisher Scientific, Cat. No. AM1560), following the manufacturer’s protocol.

### mRNA library construction

Polyadenylated transcripts were enriched from total RNA using oligo(dT)-attached magnetic beads. The isolated mRNA was then fragmented at elevated temperatures to yield appropriately sized RNA fragments. First-strand cDNA synthesis was performed using random hexamer primers and reverse transcriptase, followed by second-strand synthesis, during which dUTP was incorporated instead of dTTP to maintain strand orientation. Subsequently, the double-stranded cDNA underwent end repair, 3′-adenylation (A-tailing), and adapter ligation. Fragments of the desired size were selected, and strand specificity was ensured by digestion with the USER enzyme. Final libraries were generated through PCR amplification and purification steps. Library quality was assessed by Qubit fluorometry for concentration, real-time PCR for quantification accuracy, and fragment analysis with an Agilent Bioanalyzer. Only libraries meeting the quality criteria were pooled based on concentration and sequencing requirements and subjected to high-throughput sequencing on an Illumina platform, following Novogene’s standard workflow.

### Sequencing quality control

Raw reads (FASTQ format) were subjected to quality filtering using in-house Perl scripts. Sequences containing adapter contaminants, poly-N stretches, or excessively low-quality bases were removed. The remaining high-quality reads (referred to as clean reads) were used for all subsequent analyses. Key quality metrics, including the percentage of bases with Phred scores ≥ Q20 and Q30 and overall GC content, were calculated. All bioinformatic procedures were carried out using only the filtered reads to ensure data reliability and reproducibility.

### Read alignment to the reference genome

The Arabidopsis thaliana reference genome and annotation files (TAIR10 version) were downloaded from Ensembl Plants (https://plants.ensembl.org). Genome indexing and read alignment were performed using the splice-aware aligner HISAT2 (v2.0.5). Paired-end reads were mapped to the indexed reference genome with default settings.

HISAT2 was chosen due to its capability of integrating splice junction data from the provided annotation file, which enhances alignment accuracy and facilitates the identification of exon–intron boundaries and transcript variants.

### Gene expression quantification

Gene expression levels were estimated using *featureCounts* (v1.5.0-p3), which calculated the number of reads mapped uniquely to each annotated gene model. Raw counts were converted into FPKM (Fragments Per Kilobase of transcript per Million mapped reads), a normalisation metric that accounts for both gene length and sequencing depth, allowing for comparison across genes and samples.

While FPKM is not suitable for statistical tests of differential expression, it remains useful for descriptive analysis and visualisation of gene expression profiles.

### Differential expression analysis

Differential gene expression between experimental groups was assessed using the DESeq2 package (v1.20.0) in R. This method models count data using a negative binomial distribution, allowing for precise estimation of fold changes and statistical significance.

Raw counts from *featureCounts* served as input, and p-values were adjusted using the Benjamini–Hochberg procedure to control the false discovery rate (FDR). Genes with adjusted p-values (FDR) ≤ 0.05 were deemed significantly differentially expressed.

All differential expression analyses were performed within each genotype (WT_wound vs WT_control; *max3*_wound vs *max3*_control) to account for potential baseline expression differences.

### Transcription factor annotation and cis-regulatory analysis

Protein sequences of significantly regulated genes were retrieved from the TAIR10 dataset using the BioMart interface (https://plants.ensembl.org/biomart). These sequences were submitted to the PlantTFDB prediction tool (http://planttfdb.gao-lab.org/prediction.php) for transcription factor classification based on domain structure and sequence similarity.

Promoter regions, defined as the 1500 bp upstream of the start codon (ATG), were also retrieved using BioMart. To identify putative transcription factor binding sites (TFBS), these promoter sequences were analysed using the PlantTFDB binding site prediction tool (http://plantregmap.gao-lab.org/binding_site_prediction.php), with a significance cutoff of *p* ≤ 1e − 4.

### Gene Ontology term retrieval

To annotate the biological functions of selected *Arabidopsis thaliana* genes, Gene Ontology (GO) terms were retrieved using the Araport portal (https://www.araport.org/). A list of gene identifiers (AGI codes) was submitted to the"ThaleMine"search interface via the “Gene” data class. The output table was customised to include the associated GO terms, covering the three main categories: Biological Process, Molecular Function, and Cellular Component. The resulting data were exported in tab-delimited format (TSV) and used for downstream analysis.

## Supplementary Information

Below is the link to the electronic supplementary material.

Supplementary file1 Differentially Expressed Genes (DEGs) 3 hours after wounding (XLSX 1.53 MB)

Supplementary file2 List of wound-induced differentially expressed genes (DEGs) identified in WT and max3 (XLSX 959 KB)

Supplementary file3 Potential transcriptional factors (TFs) identified among DEGs wound-induced exclusively in WT (XLSX 28.0 KB)

Supplementary file4 Transcription factor binding site enrichment in promoters of wound-induced DEGs in WT (XLSX 7.03 MB)

Supplementary file5 Constitutive and wound-related transcriptional differences between the SL-deficient mutant max3 and WT (XLSX 2.90 MB)

Supplementary file6 Functional enrichment of WT-specific wound-responsive genes. Horizontal bar plots display the top ten GO Biological Process terms (ranked by FDR) for (A) genes up-regulated and (B) genes down-regulated exclusively in WT after wounding. Bars represent fold enrichment relative to the Arabidopsis genome background. (PPTX 288 MB)

## Data Availability

Transcriptomic data: E-MTAB-15051.
