# Supplementary material for: Strigolactones modulate jasmonate-dependent transcriptional reprogramming during wound signalling in Arabidopsis
Source: J Appl Genet. 2025 Sep 9;66(4):883–94. doi: 10.1007/s13353-025-01005-y (PMC12605546; doi:10.1007/s13353-025-01005-y)
Supplement: Supplementary file 6 — Supplementary file6 Functional enrichment of WT-specific wound-responsive genes. Horizontal bar plots display the top ten GO Biological Process terms (ranked by FDR) for (A) genes up-regulated and (B) genes down-regulated exclusively in WT after wounding. Bars represent fold enrichment relative to the Arabidopsis genome background. (PPTX 288 MB) [file 13353_2025_1005_MOESM6_ESM.pptx]

## Slide 1
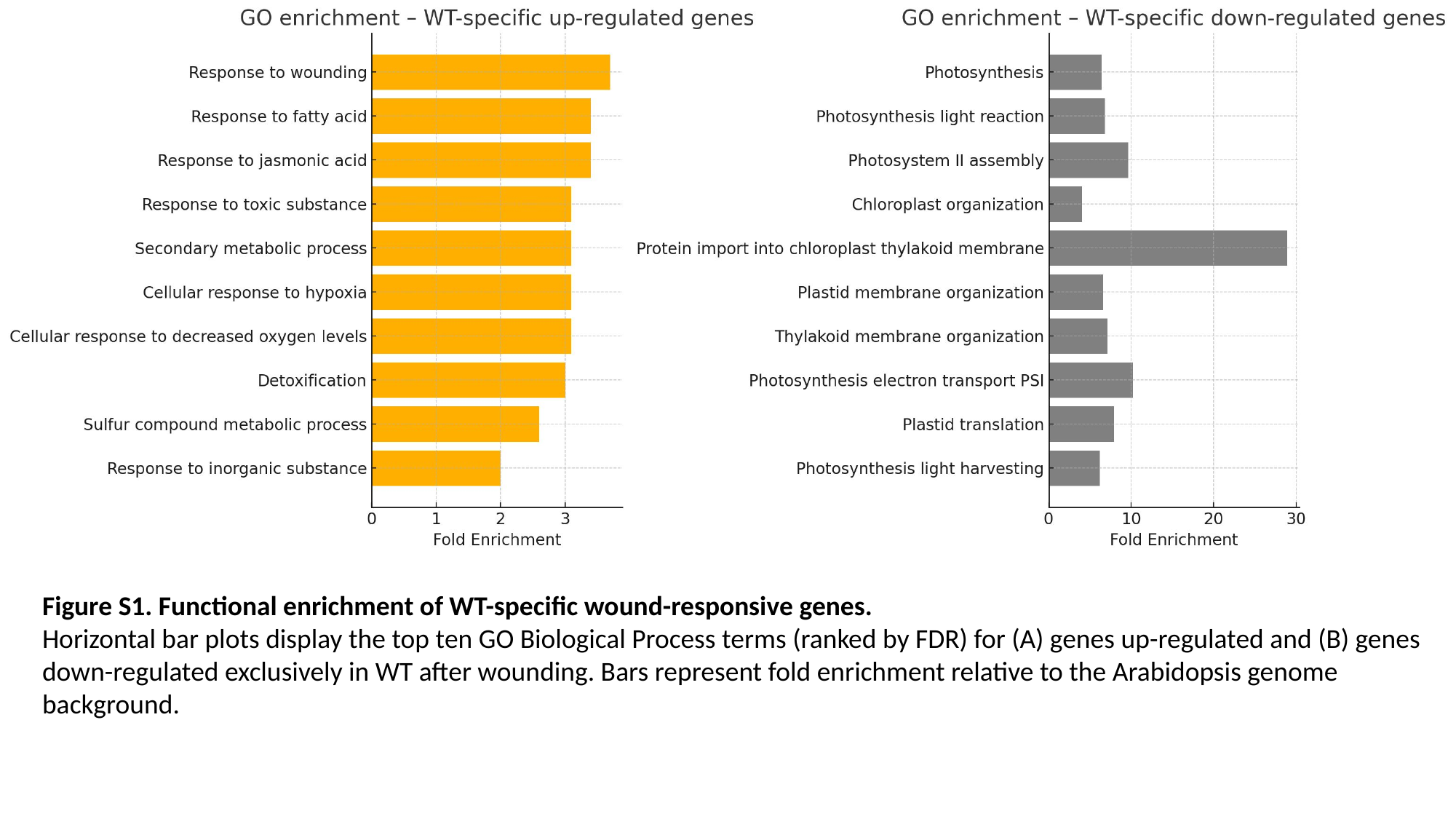

Figure S1. Functional enrichment of WT-specific wound-responsive genes.
Horizontal bar plots display the top ten GO Biological Process terms (ranked by FDR) for (A) genes up-regulated and (B) genes down-regulated exclusively in WT after wounding. Bars represent fold enrichment relative to the Arabidopsis genome background.
